# A Solid-State NMR Study of Selenium Substitution into Nanocrystalline Hydroxyapatite

**DOI:** 10.3390/ijms160511452

**Published:** 2015-05-19

**Authors:** Joanna Kolmas, Marzena Kuras, Ewa Oledzka, Marcin Sobczak

**Affiliations:** Department of Inorganic and Analytical Chemistry, Faculty of Pharmacy with the Laboratory Medicine Division, Medical University of Warsaw, ul. Banacha 1, 02-097 Warsaw, Poland; E-Mails: marzena.kuras@wum.edu.pl (M.K.); eoledzka@wum.edu.pl (E.O.); marcin.sobczak@wp.pl (M.S.)

**Keywords:** biomaterials, calcium phosphates, hydroxyapatite, selenium oxyanions, solid-state nuclear magnetic resonance, powder diffractometry

## Abstract

The substitution of selenium oxyanions in the hydroxyapatite structure was examined using multinuclear solid-state resonance spectroscopy (ssNMR). The study was supported by powder X-ray diffractometry (PXRD) and wavelength dispersion X-ray fluorescence (WD-XRF). Samples of pure hydroxyapatite (HA_300_) and selenate (HA_300_-*1.2SeO_4_*) or selenite (HA_300_-*1.2SeO_3_*) substituted hydroxyapatites were synthesized using the standard wet method and heated at 300 °C to remove loosely bonded water. PXRD data showed that all samples are single-phase, nanocrystalline hydroxyapatite. The incorporation of selenite and selenate ions affected the lattice constants. In selenium-containing samples the concentration of Se was very similar and amounted to 9.55% and 9.64%, for HA_300_-*1.2SeO_4_* and HA_300_-*1.2SeO_3_*, respectively. PXRD and ssNMR data showed that the selenite doping significantly decreases the crystallite size and crystallinity degree. ^31^P and ^1^H NMR experiments demonstrated the developed surface hydrated layer in all samples, especially in HA_300_-*1.2SeO_3_*. ^1^H NMR studies showed the dehydroxylation of HA during the selenium oxyanions substitution and the existence of hydrogen bonding in structural hydroxyl group channels. ^1^H→^77^Se cross polarization NMR experiments indicated that selenites and selenates are located in the crystal lattice and on the crystal surface.

## 1. Introduction

Since synthetic biomaterials can overcome several problems in bone grafts (*i.e.*, limited supply of autografts, risk of rejection and disease transfer of allo- and xenografts), intensive efforts have for decades been devoted to developing and improving materials such as bioceramics, polymers, metallic materials and bioglasses [1,2,3].

Calcium phosphate bioceramics, especially hydroxyapatite (HA), due to its high biocompatibility, osteoconductivity and similar composition to the inorganic fraction of mineralized tissues, play a crucial role in bone reconstructive surgery [4,5].

The hydroxyapatite crystal structure can easily host a variety of ionic substituents whose presence may affect biological and physicochemical properties [6,7,8]. Thus, such modified compounds have recently attracted much attention.

The structure of HA (Ca_10_(PO_4_)_6_(OH)_2_) is most frequently considered to be hexagonal, with space group P6_3_/m [9]. The crystallographic unit cell contains ten calcium cations arranged in two nonequivalent sites called Ca(I) and Ca(II). Calcium cations may be partially replaced by several cationic substituents, *i.e.*, Mg^2+^, Mn^2+^, Zn^2+^, Sr^2+^, Ag^+^ or Cr^3+^ [10,11]. The structural hydroxyl groups are located in columns ··· OH OH OH ···, where oxygen atoms are too distant (3.44 Å) to form hydrogen bonds [9]. The OH groups in columns are frequently replaced by various monovalent and bivalent anions: Cl^−^, F^−^ and CO_3_^2−^, O^2−^, S^2−^, respectively [12,13]. The tetrahedral trivalent orthophosphates PO_4_^3−^ may be substituted not only by other trivalent (*i.e.*, AsO_4_^3−^) but also by bivalent (*i.e.*, CO_3_^2−^) and tetravalent (SiO_4_^4−^) anions [14,15]. It is important to note that all substitutions may be the source of lattice distortion, vacancies, crystal defects or hydrogen bonds in OH groups’s columns, which in turn play a significant role in HA crystal size and morphology [6,8].

Inspired by previous research on hydroxyapatite enriched in various ions, we have decided to study the incorporation of selenium oxyanions (selenate SeO_4_^2−^ and selenite SeO_3_^2−^) into the HA crystal structure.

Selenium is an important element in bone physiology. It plays a crucial role in various metabolic processes as a constituent of a great number of enzymes [16]. Its deficiency may retard growth of bones and increase the risk of bone disease (*i.e.*, osteopenia, osteoarthritis and osteoporosis) [17,18]. Selenium, as a constituent of selenoproteins, plays a significant role in the proliferation of osteoblasts and osteoclasts. It is also important to note that selenium has shown considerable promise as an anticancerogenic agent [19].

In our previous work [20] we have focused on the synthesis of hydroxyapatite containing selenite or selenate ions at various concentrations. It has been shown that nanocrystalline, selenium-enriched HA materials may be prepared by conventional wet method.

The main objective of this work is the structural characterization of hydroxyapatite substituted with selenium oxyanions. We present here a detailed solid-state nuclear magnetic resonance (ssNMR) investigation on ^77^Se, ^1^H and ^31^P nuclei.

## 2. Results and Discussion

### 2.1. Powder X-ray Diffraction Analysis

**Figure 1:**
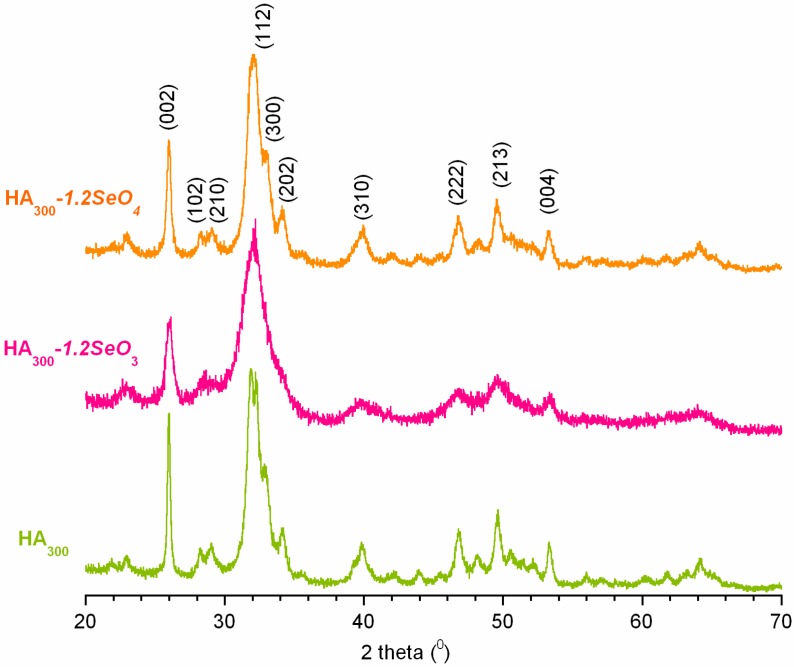
PXRD patterns of the analysed samples: HA_300_, HA_300_*-1.2SeO_3_* and HA_300_*-1.2SeO_4_*.

Figure 1 presents the powder X-ray diffraction patterns of pure hydroxyapatite (HA_300_) and selenium- containing samples (HA_300_-*1.2SeO_3_* and HA_300_-*1.2SeO_4_*). Each diffractogram matches the International Centre for Diffraction Data (ICDD) standard diffractogram of HA (No. 9.432) well. The results indicate that no other crystalline phase was detected. All the powders exhibit relatively broad reflection lines, especially the HA_300_-*1.2SeO_3_* sample (see Figure 1). These results indicate that the crystallites are fine and the crystallinity degree is low [21].

We have calculated the crystallites’ sizes according to Scherrer’s formula:

(1) $$D=\frac{0.94\times \text{λ}}{{\text{β}}_{1/2}\times \cos\text{θ}}$$

where *D* is a domain size (crystallite size in nm), λ is the wavelength of the radiation (in nm), β_1/2_ is the peak full-width at half- maximum (in radians) and θ is the diffraction angle of the corresponding reflex.

It was confirmed that all the powders are nanocrystalline with an average crystallite size (in nm) decreasing in the series: HA_300_ (18) > HA_300_-*1.2SeO_4_* (15) > HA_300_-*1.2SeO_3_* (9).

In order to estimate the crystallinity degree of the obtained powders the formula below was used [22]:

(2) $${\text{χ}}_{\text{c}}={\left(\frac{K}{{\text{β}}_{\left(002\right)}}\right)}^{3}$$

where χ_c_ is the crystallinity degree corresponding to the fraction of crystalline phase in powder, *K* is a constant. (*K* for a great number of hydroxyapatites is 0.24), β_(002)_ is the peak (002) full-width at half- minimum (in degrees).

The calculations have shown that the crystallinity degree dramatically decreases in the series: HA_300_ (0.7) > HA_300_-*1.2SeO_4_* (0.5) >> HA_300_-*1.2SeO_3_* (0.1).

Table 1 collects the lattice parameters obtained from Rietveld refinements of the PXRD data. These data show that the incorporation of selenite or selenate ions into hydroxyapatite structure affected the lattice constants; however, SeO_3_^2−^ substitution causes higher increments of both *a* and *c* constants. These data are in good agreement with our previous measurements described in [20].

**Table 1:** Various parameters of the studied samples. The unit cell parameters *a* and *c* (in Å), crystallinity index and crystallite size (in nm) were calculated from the PXRD patterns using Rietveld analysis. Chemical composition of the samples was studied using WD-XRF method.

Characteristics	HA_300_	HA_300_-*1.2SeO_3_*	HA_300_-*1.2SeO_4_*
**Cell parameters (Å)**	*a* = 9.4287 *c* = 6.8772	*a* = 9.5084 *c* = 6.8891	*a* = 9.4333 *c* = 6.8879
**Crystallite size (nm)**	18	9	15
**Crystallinity index**	0.7	0.1	0.5
**Se content (wt %)**	-	9.64	9.55
**Ca/(P + Se)**	1.62	1.56	1.60

The results of WD-XRF elemental analysis are reported in Table 1. The selenium contents are similar to those of the corresponding amount of starting materials. This may be proof of the incorporation of selenite and/or selenate into the crystal lattice. It should be noted that these ions are not eliminated, even through intensive rinsing of the powder with distilled water.

### 2.2. ^31^P Solid-State NMR Spectroscopy

^31^P solid-state NMR spectra were acquired for all samples using two different techniques: the one-pulse (BD) and ^1^H→^31^P cross polarization (CP) techniques. Briefly, the BD signals come from all phosphorus-31 nuclei, while CP lines may be assigned to the ^31^P nuclei located close to protons (in the case of hydroxyapatite, close to structural hydroxyl groups and to molecules of water in the crystal structure or adsorbed on the crystal surface).

**Figure 2:**
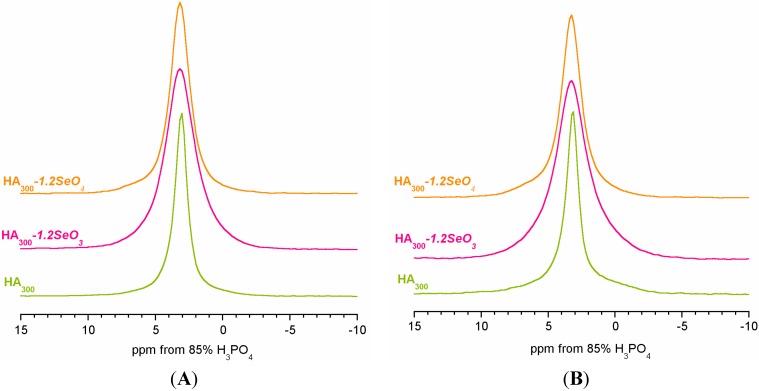
^31^P BD (**A**) and CP (**B**) MAS NMR spectra of the analysed samples.

All the samples give one signal at about 3 ppm in BD as well as CP experiments; that is a characteristic feature of apatites [23,24] (see Figure 2A,B and Table 2).

**Table 2:** Curve fitting results for the ^31^P CP NMR spectra (MAS at 7.0 kHz, the CP contact time of 2 ms). The peaks have been assigned according to Pajchel *et al.* [25].

Peak Characteristics	HA_300_		HA_300_-*1.2SeO_3_*		HA_300_-*1.2SeO_4_*	
	Narrow	Broad	Narrow	Broad	Narrow	Broad
Chemical shift (ppm)	3.16	3.44	3.26	3.31	3.17	3.38
FWHM ^a^ (Hz)	159	897	332	879	247	867
LF ^b^	0.5	0.0	0.6	0.1	0.6	0.0
% of total area	76	24	49	51	66	34
**Narrow line characteristics**						
FWHM ratio: CP/BD	0.95		0.83		0.92	
Area ratio ^c^: CP/BD	0.66		0.40		0.57	

^a^ Full width in half minimum; ^b^ Lorentzian fraction; ^c^ Measured at ν_MAS_ = 0 kHz.

Both BD and CP signals are significantly broader for the HA_300_-*1.2SeO_3_* sample. The FWHM parameter (shown in brackets in Hz) measured for BD spectra increased in the following order: HA_300_ (165) < HA_300_-*1.2SeO_4_* (267) << HA_300_-*1.2SeO_3_* (405). Since the degree of crystallinity of the apatitic samples is reflected in the width of the NMR line, it may be assumed that incorporation of selenium oxyanions, especially selenite into the crystal lattice leads to disorder in the hydroxyapatite structure.

In all the samples, the CP lines are slightly wider than the corresponding BD lines and the FWHM value amounts to 176, 270 and 420 Hz for HA_300_, HA_300_-*1.2SeO_4_* and HA_300_-*1.2SeO_3_*, respectively.

**Figure 3:**
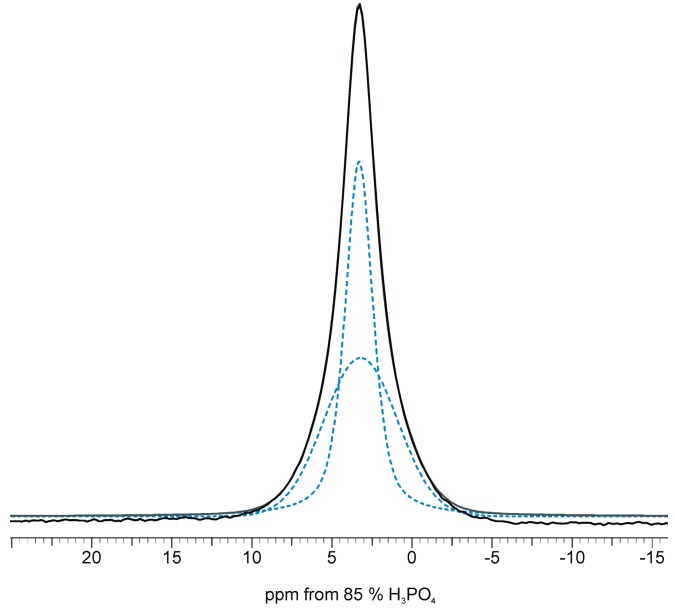
Representative peak fittings of the ^31^P CP NMR spectrum for the HA_300_*-1.2SeO_3_* sample.

Curve fitting of the ^31^P CP NMR spectra have disclosed the presence of two overlapped signals: a narrow one located on the top of the significantly broader one (see Figure 3 and Table 2). It should be noted that the BD line is also a composite signal where the broad signal is very weak (data not shown). Consider that BD lines come from all ^31^P nuclei, located on the surface or/and in the crystal lattice at whatever distance from protons. Thus, the signals of phosphorus-31 nuclei located close to protons are relatively weaker than in CP spectra.

According to Pajchel *et al.* [25] the narrow line in the CP experiment is generated from ^31^P nuclei located close to protons of structural hydroxyl groups from the crystal interior. The broad signal may be assigned to the phosphorus-31 nuclei located close to a water-rich environment, and hence especially to the ^31^P from the surface hydrated layer.

The broad lines are mostly Gaussian in shape, as is characteristic for disordered and mobile systems [25]. The amount of phosphorus from the surface hydrated layer is relatively larger in the HA_300_-*1.2SeO_3_* sample than in HA_300_ and HA_300_-*1.2SeO_4_*. PXRD results have shown that the samples containing selenite ions have the smallest crystallites thus we can assume that the specific surface area of this sample is the largest. Therefore, the HA_300_-*1.2SeO_3_* sample may exhibit the most developed surface hydrated layer. Due to the hydroxyapatite powder’s tendency to agglomerate, the surface hydrated layer remains, even under heating at 300 °C during 2 h.

We have examined the cross-polarization efficiency according to the method described in [26]. In order to avoid the influence of rotation on the CP process, the ^31^P (CP and BD) were acquired in the static condition. The CP/BD intensity ratios were calculated, where intensity was measured as a peak area. The results show that in all the spectra the CP signal is significantly weaker in comparison with the BD signal. Our previous study presented in [27] suggested that the signal attenuation is strongly dependent on the surface hydrated layer and on the concentration of the structural hydroxyl groups.

### 2.3. ^1^H Solid-State NMR Spectroscopy

**Figure 4:**
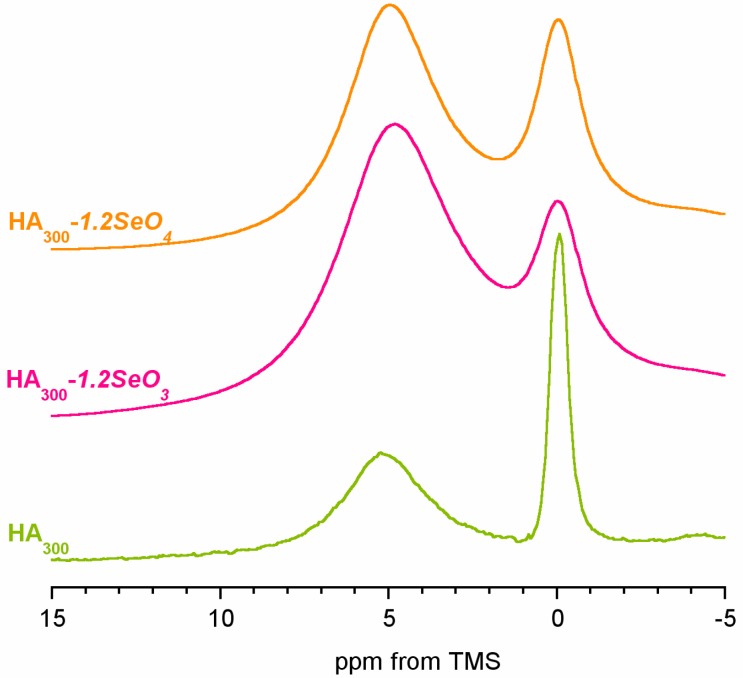
The ^1^H BD NMR spectra of HA_300_, HA_300_*-1.2SeO_3_* and HA_300_*-1.2SeO_4_*.

^1^H MAS NMR spectra of the samples are presented in Figure 4. All spectra are similar and contain two lines at *ca.* 0 and 5.4 ppm. According to previous studies, the line at *ca.* 0 ppm originates from the structural hydroxyl groups [23,25,28]. It should be pointed out that stoichiometric hydroxyapatite structural OH groups are located in columns in such a way that the successive oxygen atoms are too distant for hydrogen bonding formation. Thus, the OH line in pure hydroxyapatite occurs at *ca.* 0.0 ppm. In our spectra the chemical shift of this line increases in the following order (ppm value in brackets): HA_300_ (0.03) < HA_300_-*1.2SeO_4_* (0.19) < HA_300_-*1.2SeO_3_* (0.33). Simultaneously, significant broadening of the OH line in the spectra of samples containing selenium is observed (see Figure 4). Therefore, it may be assumed that selenium’s incorporation into the hydroxyapatite structure induces a high disorder in the OH columns and the formation of weak hydrogen bonds [29]. It should be considered that the substitution of bivalent selenium oxyanions (selenate or selenite) for trivalent orthophosphate causes the formation of positively charged vacancy that should be compensated. The mechanism of compensation proposed in [20] and parallel with that in carbonate type B substitution, consists in the joint release of calcium cation and hydroxyl anion. For this reason, we have calculated the hydroxyl groups’ content in the samples with reference to stoichiometric hydroxyapatite [30].

**Figure 5:**
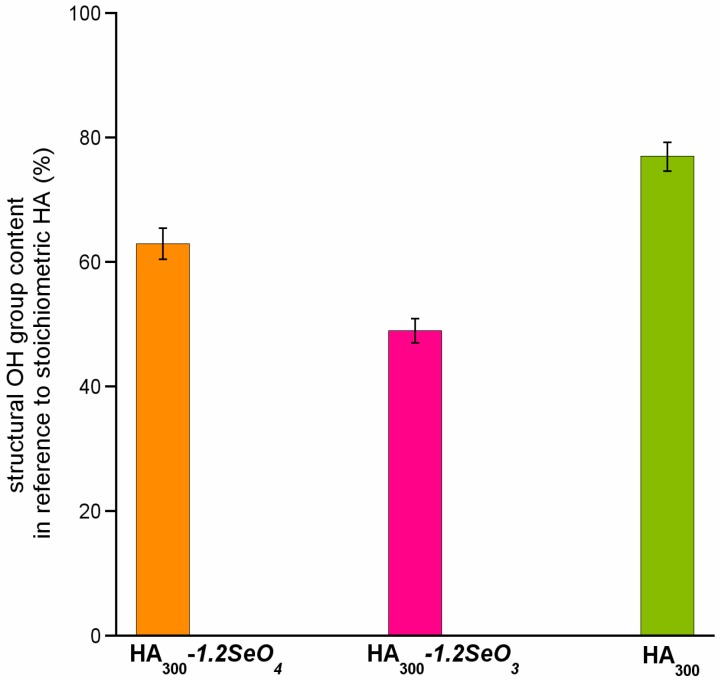
Structural hydroxyl groups content in reference to stoichiometric hydroxyapatite (%).

The results are shown in Figure 5. We have found the highest concentration of structural hydroxyl groups in the HA_300_ sample (77%). The introduction of selenium oxyanions into the hydroxyapatite structure causes an important decrease of OH content (63% and 49% of structural OH groups for samples containing selenate and selenite, respectively).

The problem of the structural hydroxyl groups’ content both in synthetic and biological apatites has been widely discussed in the literature. In [25] it has been shown that in nanocrystalline hydroxyapatite the content of structural OH groups decreases with the decreasing of crystal size. Nanocrystalline apatite, beside the well-ordered core of the crystals, has a complex surface hydrated layer which interacts with the apatitic core. Moreover, it was found that the disorder in OH columns in nanocrystals may be caused by an uptake of structural water [25,31].

It may be concluded that in the studied samples that are nanocrystalline, the decrease of the OH groups’ content probably involves not only the release of hydroxyl ions during the substitution but also the decreasing crystal size and the presence of water in the OH columns.

The signal at *ca.* 5.4 ppm is typically assigned to hydrogen bonded water molecules. According to the literature, this line is composite and may comprise both water adsorbed on the surface and structural water located in the OH columns and forming hydrogen bonds among themselves and with OH groups [23,32]. However, the contribution of the structural water signal is very low. Water molecules from the surface may have diverse nature. First and foremost, they are components of the surface hydrated layer, a characteristic feature of uncalcified hydroxyapatite. Moreover, a similar chemical shift may be attributed to loosely bonded water, present in “raw”, unheated hydroxyapatite crystals. In the HA_300_, HA_300_-*1.2SeO_4_* and HA_300_-*1.2SeO_3_* samples, when heated at 300 °C, loosely bonded water is practically removed, and that is why the lines at *ca.* 5.4 ppm originate from water existing in the surface hydrated layer.

### 2.4. ^77^Se Solid-State NMR Spectroscopy

Solid-state ^77^Se NMR was used to monitor selenium oxyanions in the HA_300_-*1.2SeO_4_* and HA_300_-*1.2SeO_3_* samples. It should be noted that natural abundance ^77^Se NMR studies are quite challenging due to unfavourable nuclear properties. Despite nuclear spin −1/2, 7.58% natural abundance and large chemical shift range (~3000 ppm), ^77^Se exhibits extremely long spin-lattice relaxation time [33]. For that reason it was very difficult to obtain ^77^Se NMR spectra using a standard one-pulse (BD) experiment. To overcome the above-mentioned challenge, we performed a cross-polarization experiment from protons to ^77^Se nuclei. This way, the relaxation time was significantly shortened and the signal was enhanced due to spin magnetization transfer.

**Figure 6:**
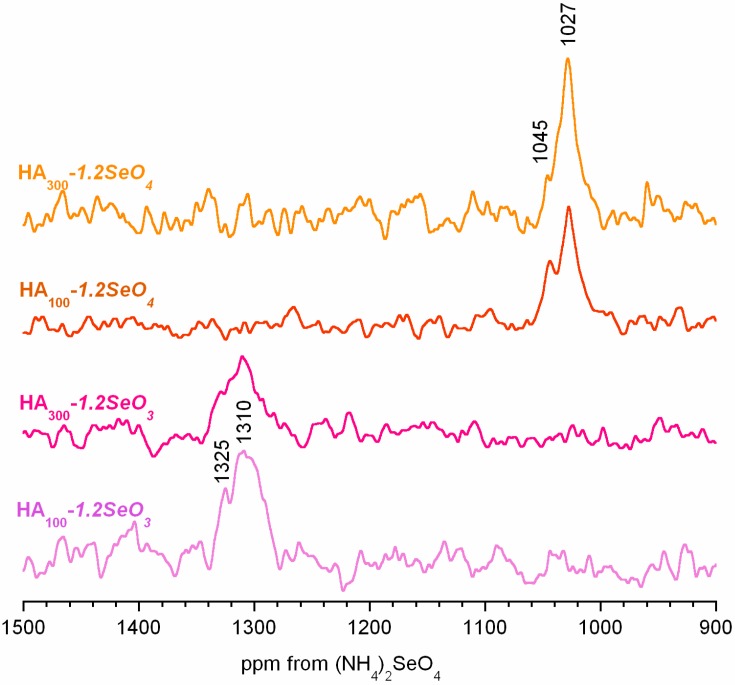
The ^1^H→^77^Se CP NMR spectra for the selenium containing samples. Samples of HA_100_*-1.2SeO_3_* and HA_100_*-1.2SeO_4_* were dried at 100 °C.

The HA_300_-*1.2SeO_4_* and HA_300_-*1.2SeO_3_* spectra are shown in Figure 6. Both spectra are very noisy in spite of the long accumulation time (20 h). They exhibit similar broad and poorly resolved signals with distinguishable left-side minor peaks.

The isotropic chemical shift ranges are significantly different for the spectra. The ^77^Se CP MAS NMR main signals occur at 1027 and 1310 ppm for HA_300_-*1.2SeO_4_* and HA_300_-*1.2SeO_3_*, respectively, whereas minor peaks may be found at *ca.* 1045 and 1325 ppm, respectively. According to the literature, ^77^Se δ_iso_ for inorganic selenates and selenites are generally found in the 1024–1050 ppm and 1257–1300 ppm ranges, respectively [33,34]. Thus, we can assume that in the HA_300_-*1.2SeO_4_* spectrum both signals originate from selenates and that in the HA_300_-*1.2SeO_3_* spectrum the lines come from selenite ions. However, their interpretation is not trivial. We believe that the lines at *ca.* 1027 ppm in HA_300_-*1.2SeO_4_* (and *ca.* 1306 ppm in HA_300_-*1.2SeO_3_*) may be assigned to selenates (and selenites) from the hydroxyapatite interior (selenium oxyanions incorporated into the crystal lattice). The minor lines at *ca.* 1044 and 1325 ppm, respectively may be attributed to selenates or selenites from the hydrated surface layer. In order to support this interpretation, we performed a ^77^Se CP MAS experiment for the samples heated at 500 °C (for 2 h). The spectra are presented in Figure 6. Consider that the minor lines are relatively weaker than in the samples heated at 300 °C. Such species first and foremost cross-polarize from protons of water from the surface hydrated layer. During heating, the water is gradually removed and the cross-polarization process is impossible. We are aware that this interpretation is tentative and should also be supported by ^77^Se MAS BD and ^77^Se CP MAs experiments with various contact times (CP kinetics). However, it requires enrichment of samples in selenium-77 nuclei.

## 3. Experimental Section

### 3.1. Preparation of Samples

Samples of pure and selenium-enriched hydroxyapatite were prepared by the coprecipitation method using calcium nitrate tetrahydrate, diammonium hydrogen phosphate and optionally natrium selenite pentahydrate or natrium selenate as the sources of calcium, phosphorus and selenium (IV) or selenium (VI), respectively [20].

Briefly, a mixture of (NH_4_)_2_HPO_4_ and Na_2_SeO_4_ or Na_2_SeO_3_·5H_2_O dissolved in distilled water was added dropwise to Ca(NO_3_)_2_·4H_2_O solution. The temperature of the reaction was set to 50 °C and the pH was adjusted in the 8–9 range by means of a concentrated solution of ammonia.

The precipitates were aged in the mother liquor for 24 h, decanted, and washed with distilled water. After repeating the washing procedure, the white powders were dried overnight in air at 100 °C. Finally, the powders were heated at 300 °C for 2 h.

Hereafter, samples of pure HA and those containing selenates or selenites are designated HA_300_, HA_300_-*1.2SeO_3_* and HA_300_-*1.2SeO_4_*, respectively.

### 3.2. Characterization

The phase composition of the samples was evaluated by powder X-ray diffraction (PXRD, Bruker D8 Discover diffractometer, Karlsruhe, Germany). The measurements were carried out using CuKa radiation (λ = 1.54 Å) over the 2θ range of 20°–45°, using a step size of 0.024°, step time 4 s and locked coupled (theta–theta) geometry. The lattice parameters were estimated using TOPAS software (version 3, Bruker).

The content of selenium in hydroxyapatites enriched with selenium oxyanions was measured using wavelength dispersion X-ray fluorescence (WD-XRF) after dissolution in HNO_3_.

Solid-state nuclear magnetic resonance (ssNMR) spectra were recorded using a Bruker Avance 400 WB spectrometer at proton, ^31^P and ^77^Se resonance frequencies of 400, 160 and 76 MHz, respectively. A 4 mm diameter, Bruker double-bearing magic angle spinning (MAS) probe and zirconia rotors, driven by dry air, were used. All measurements were performed at room temperature.

The ^1^H Bloch-decay spectra were acquired under a MAS rate at 12 kHz, with 32 transients and a long recycle delay (50 s). For estimation of structural hydroxyl groups content, we used the procedure described in [30]. The spectrum of each sample was recorded six times. The probe background was carefully subtracted. The spectra were externally referenced to tetramethylosilane (TMS).

The ^31^P ssNMR spectra, referenced to an 85% H_3_PO_4_, were performed using both: conventional one-pulse (Bloch-decay, BD) and cross-polarization (CP) pulse sequences. The MAS rate was set at 7 kHz. Thirty-two transients were accumulated with a contact time of 2 ms for CP and a recycle delay of 10 and 30 s for CP and BD experiments, respectively. The ^31^P NMR spectra were acquired at ν_MAS_ = 7 kHz as well as under static conditions.

For the samples containing selenium, the ^1^H→^77^Se CP MAS spectra were recorded at 5 kHz, with a contact time of 3 ms, a recycle delay of 1 s and a number of scans of 40,000. The ^77^Se NMR spectra were referenced to (NH_4_)_2_SeO_4_.

## 4. Conclusions

The incorporation of selenium into nanocrystalline hydroxyapatite structure was investigated by solid-state NMR on two different samples containing selenite and selenate. The main conclusions of this work may be summarized as follows:

- The content of selenium in HA_300_-*1.2SeO_4_* and HA_300_-*1.2SeO_3_* samples is similar and amounts to 9.55% and 9.64%, respectively.
- The analysed samples are nanocrystalline hydroxyapatites and do not contain other crystalline phases.
- The selenite and selenate incorporation has a significant impact on the crystal lattice parameters, crystallite size and crystallinity degree.
- The incorporation of selenium oxyanions causes the decrease of structural hydroxyl groups’ content.
- Selenite and selenate ions are located in the hydroxyapatite crystal interior and on the crystal surface in the surface hydrated layer.
